# Incongruence in body image and body mass index: A surrogate risk marker in Black women for type 2 diabetes mellitus

**DOI:** 10.4102/phcfm.v5i1.496

**Published:** 2013-07-18

**Authors:** Rynal Devanathan, Viveka Devanathan, Tonya M. Esterhuizen

**Affiliations:** 1 Department of Family Medicine, University of KwaZulu-Natal, South Africa; 2 R.K. Khan Hospital, Chatsworth, South Africa; 3 Programme of Biostatistics, University of KwaZulu-Natal, South Africa

## Abstract

**Background:**

Excess weight contributes to the development and progression of Type 2 diabetes mellitus (T2DM). Distorted body image amongst urban Black women and the perception that thinness is linked with HIV, may however be compounding the problem, particularly in areas with a high HIV burden.

**Objectives:**

This study aimed to compare the perception of body image in urban Black women with and without T2DM.

**Methods:**

A cross-sectional comparative study was conducted on 328 Black women systematically sampled into two groups (with and without T2DM). Body mass index (BMI) (weight [kg]/height [m^2^]) was determined and the adapted Stunkard Body Image Silhouettes for Black women was used to determine perceived body image (PBI).

**Results:**

Seventy-two per cent had T2DM and in this group 89% were obese, with a mean BMI of 39.5 kg/m^2^ (s.d. ± 8.5). In the non-diabetes group (NDG) 44% were obese, with a mean BMI of 31.3 kg/m^2^ (s.d. ± 9.0) Black women underestimated their body image across all weight categories (*p* < 0.05). Both groups (99% of the study group) also perceived thinness as being associated with HIV.

**Conclusions:**

This study identified an incongruence between PBI and actual BMI amongst urban Black women. This, combined with their belief that thinness is associated with HIV, places those with T2DM at risk of secondary complications arising from diabetes mellitus, and those without diabetes mellitus at a higher risk of developing T2DM. A discrepancy between PBI and BMI may therefore serve as a risk marker to alert clinicians to use a more ethno-cultural specific approach in engaging with urban Black women regarding weight loss strategies in the future.

## Abstrait

**Incongruence l'image corporelle et l'indice de masse corporelle: Un marqueur de risque de substitution chez les femmes noires de diabète de type 2**

**Contexte:**

La surcharge pondérale contribue au développement et à la progression du diabète non insulo-dépendant de type 2. La perception déformée du corps de la femme chez les femmes noires et la perception que la minceur est associée au VIH pourraient encore aggraver ce problème, notamment dans les zones affectées par une forte prévalence d'infection par le VIH.

**Objectif:**

L'objectif de cette étude était de comparer la perception de l'image du corps de la femme chez les femmes noires vivant en milieu urbain et ne souffrant pas de diabète non insulo-dépendant de type 2.

**Méthode:**

Une étude comparative transversale a été réalisée auprès de 328 femmes noires échantillonnées de manière systématique dans deux groupes (avec et sans diabète non insulo-dépendant de type 2). L'indice de masse corporelle (IMC) (poids (kg)/taille (m) a été calculé et l’échelle de Stunkard des silhouettes corporelles adaptée aux femmes noires a été utilisée afin de déterminer l'image corporelle perçue.

**Résultats:**

Soixante-douze pour cent des femmes souffraient de diabète non insulo-dépendant de type 2 et dans ce groupe, 89% étaient obèses, avec un IMC moyen de 39,5 kg/m (ET ± 8,5). Dans le groupe de femmes non diabétiques, 44% étaient obèses, avec un IMC moyen de 31,3 kg/m (ET ± 9). Les femmes noires sous-estimaient leur image corporelle dans toutes les catégories de poids (*p* < 0.05). Chacun des groupes (99% du groupe de l’étude) percevait également la minceur comme étant associée au VIH.

**Conclusions:**

Cette étude a permis d'identifier une dissociation entre image corporelle perçue et IMC réel chez les femmes noire vivant en zone urbaine. Ceci, combiné à leur conviction que la minceur est associée au VIH, fait courir aux femmes souffrant de diabète non insulo-dépendant de type 2 le risque de souffrir de complications secondaires associées au diabète non insulo-dépendant, et à celles ne souffrant pas de diabète un risque plus élevé de développer un diabète non insulo-dépendant de type 2. Une incohérence entre image corporelle perçue et IMC peut par conséquent être utilisée comme marqueur de risque incitant les médecins à utiliser une approche davantage ethnoculturelle pour promouvoir des stratégies de perte de poids chez les femmes noires vivant en milieu urbain à l'avenir.

## Introduction

### Background

Global mortality during 2008 was estimated to be 57 million, with 63% being attributed to chronic diseases of lifestyle, primarily circulatory diseases, diabetes mellitus (DM), cancer and chronic lung diseases.^1^ An alarming 29 million deaths (80%) took place in emerging socio-economic countries, including South Africa.^1^ The occurrence of non-communicable diseases (NCDs) is growing fast and is currently projected to cause almost 75% as many deaths as infectious, maternal, perinatal, and nutritive diseases by 2020, and to surpass them as the most frequent origin of mortality by 2030.^2, 3^

The main determinants of NCD-related deaths in 2008 were due to circulatory diseases (56%), cancers (25%), and lung diseases including asthma and chronic obstructive airway disease (14%), with DM causing an additional 4% of deaths.^1^ There are currently 346 million people worldwide living with DM, of which 80% are living in low to middle socio-economic countries. In 2000, the WHO projected that the mortality due to DM will double between 2005 and 2030 with 814 000 people in South Africa having the condition, and this number is projected to rise to 1.3 million by 2030 if no intervention strategies are put in place.^4, 5^

### The South African setting

South Africa (SA) currently faces a quadruple burden of disease which includes poverty-related conditions, NCDs, injuries and HIV. The mortality owing to NCDs in the country was estimated at 37% in 2000 and the socio-economic transition in the Black population was implied to contribute to NCDs.^6^ Obesity as a modifiable risk factor for developing Type 2 DM (T2DM) is a major public health dilemma in SA where, according to the 2003 South African Demographic and Health Survey (SADHS), a third of the men and more than half of the women had a Body Mass Index (BMI) of ≥ 25 kg/m.^2, 7^ Obesity figures (BMI ≥ 30 kg/m^2^) are particularly high amongst the female populace, with an estimated prevalence of between 31% and 34%.^7, 8, 9^

The belief that was once held, that obesity was not associated with health hazards in the SA Black population, has been challenged by evidence from large cohort studies such as the Nurses’ Health Study which demonstrated that the threat of T2DM escalated by 40 times with a BMI of 22 kg/m^2^ – 35 kg/m.^2, 10, 11^ The determinants of the obesity epidemic and the subsequent sequel of T2DM in SA are not straightforward. Significant contributory factors to the obesity statistics in SA include the accessibility of cheap unhealthy foods and barriers to physical activity, which are further enhanced by urbanisation.^12^

Recently a women's perception of her body image has been explored as a risk factor for obesity.^13, 14^ Body image is defined as the personal appreciation individuals hold regarding their physiques; it embraces attitudes and self-perception about their body size. These opinions are thoughts held in the minds of individuals about how they are regarded by others. A large study by Bays et al.^15^ Recently concluded that misperception of body image may have preceded T2DM in a United States of America (USA)-based population. Beliefs and attitudes of Black women toward body weight may play a significant role in the high prevalence of obesity in SA.^12^ Based on the findings of the SADHS 2003^7^, Puoane et al.^12^ Reported that although obesity was highest in the Black population of SA, obese Black women tended not to perceive themselves as such (the survey showed that although 57% in fact had a BMI greater than 25 kg/m^2^, only 21% self-reported being overweight or obese).^7^

### The ‘Big is Beautiful’ study

The ‘Big is Beautiful’ study^16^ conducted in the large urban township of Khayelitsha, Cape Town, rendered insight into the socio-cultural issues that Black women in SA are facing. This study found that female community health workers, who were supposed to lead by example, preferred an overweight shape which they associated with dignity, respect, confidence and beauty, thus rendering a mixed message to their audiences. These women's perception of an ideal weight appeared to be influenced by various factors, with culture playing a major role. The study found that, for Black women, being overweight and even obese had many positive connotations. The women thought that being overweight or obese makes a woman more desirable to men, signifies that her husband is taking good care of her and shows that she is ‘able to stir big pots and would not be blown away by the strong Cape winds’. By comparison, being thin was paralleled with unhappiness, ill-treatment and, most importantly, with having HIV.^16^ Matoti-Mvalo and Puoane^14^ further reported that 69% of these urban Black women linked being thin to being infected with HIV or having AIDS, that 34% preferred being overweight and that 31% associated being overweight with good health. These findings exemplify the fact that a big body size is more acceptable in this population, whilst being thin is associated with HIV. These factors may cause this population of Black women to be reluctant to lose weight for fear of stigmatisation.^14^

### Objectives of this study

SA presently faces a formidable task, having to deal with an escalating HIV prevalence as well as with the spiralling obesity epidemic with associated T2DM. By 2008, it was estimated that 5.6 million South Africans were seropositive for HIV, with KwaZulu-Natal (KZN) being the hardest-hit of the nine provinces with a prevalence reaching 16%.^17^ There is a paucity of research addressing incongruence in body image and BMI as a possible risk marker for T2DM in urban Black women living in an area of high HIV burden. The Word Health Organization (WHO) and the European Union stressed the significance of addressing the ethnosocio-cultural and structural–physical impacts for the successful prevention and treatment of the obesity pandemic as part of addressing the global rise in T2DM.^18, 19^ It is important in a South African setting that the perceptions of Black women regarding their body image be explored when planning intervention strategies to stem the tide of obesity and associated T2DM. This study aimed to compare the perceptions of body image amongst urban Black women with and without T2DM in KZN, SA, in order to establish whether incongruence in body image and BMI might be a surrogate risk marker for the development of T2DM in these Black women. This was done by determining the prevalence of obesity in both groups, comparing their body image perceptions, and comparing their perceptions in relation to preferred, healthy, and diseased (e.g. HIV) body image profiles. This sets the platform for further research in developing a more culture-specific approach to weight loss in this complex socio-cultural group.^16^

## Research method and design

### Selection of patient sample

A comparative cross-sectional study was carried out at Wentworth Hospital, a district hospital in Durban, KZN. All Black women in the age range of 18–70 years who frequented the NCD clinic during the period June 2010 – October 2010 were selected as a convenience sample. From the chronic patient booking register it was estimated that approximately 2000 Black women attended the NCD clinic in a year. A sample of approximately 10% (*n* = 328) will allow a 5% margin of error in estimating categorical population parameters with 95% confidence.^20^ Every sixth woman attending the NCD clinic was systematically sampled for participation in the study if she gave consent. If a women did not consent, then the next woman was selected and the reasons for non-participation were recorded in the case record file (CRF). The CRF was designed to capture information regarding demographic data, chronic medical conditions, body weight, height and chosen body image silhouettes.

### Procedure

The women were then divided into two groups on the basis of pre-existing T2DM. The final sample included 237 with T2DM, and 91 without the condition. Black women with type 1 diabetes or gestational DM were not included in the study. Each case record was completed by the principal investigator so as to ensure reliability of the results.

Height was measured using a metal measuring tape, with the study participants dressed in a thin hospital gown without shoes and headgear so as to ensure correct height estimations. The measurements were rounded off to the nearest 0.1cm. The body mass was ascertained in the same clothing using a standardised automated numerical load-cell scale with a ceiling mass of 136 kg (Precision Health Scale – UC, correct to 0.05 kg). An equivalent measure was used for those who weighed more than 136 kg (Soehnle Medica, maximum 150 kg, accurate to 0.5 kg). For those subjects weighing more than 150 kg, a single foot was placed on each of two equivalent scales.^16^ The same scale was used throughout the study in order to ensure reliability of the results. The expressed kg/m^2^ of the BMI was determined and logged in the CRF.

### Perceived body image

To determine the perception of the woman regarding her own body image, the Stunkard Body Silhouettes were used. These images of body size were proposed by Stunkard et al.^12^ as an easy tool to determine perception of one's body figure and have been authenticated and adapted for use in the South African context.^21, 22^ This method was selected to ensure that women who are illiterate were able to express their perceptions of body image in order to reduce possible selection bias.^21^ The study participants were required to select the silhouettes that very much resembled (1) their perception of themselves, (2) a ‘preferred’ body image, (3) a healthy body image, and (4) an image of one who has HIV. The participants were asked in each instance to choose from body images 1–8 with the first silhouette representing the thinnest, and the eighth the largest body image. The body image chosen was recorded in the CRF.

### Analysis

The data was entered into a Microsoft Excel^®^ spreadsheet which was then exported into the SPSS-16 statistical package for further analysis, conducted by the Department of Biostatistics, University of KwaZulu-Natal (UKZN). Standard descriptive statistics (numbers, proportions and means) and standard deviations were used to describe the study group and their choice of silhouettes representing their body image. The difference in proportions between the perceived body image (PBI) and actual BMI was tested for significance by means of the Chi-square test and was set a priori as *p* < 0.05.

## Ethical considerations

The Biomedical Research Ethics Committee of the Faculty of Health Sciences, UKZN, permitted the study. The Chief Executive Officer of Wentworth Hospital and the KZN Provincial Department of Health approved the study. Participation in the study was voluntary and issues of confidentiality were assured. The HIV status of the individuals was not requested as this was not part of the study objective.

## Results

A total of 328 Black women who attended the NCD clinic at Wentworth Hospital during the study period participated in the study. Of these women, 72% had T2DM. The ages ranged from 19 to 70 years with the diabetic group (DG) (average of 54 years [s.d. ± 9.8]) being older than the non-diabetic group (NDG) (average of 39 years [s.d. ± 12.1]). The DG had a higher mean BMI – 39.5 kg/m^2^ (s.d. ± 8.5), which was higher that of the NDG (31.3 kg/m^2^ [s.d. ± 9.0]) – but both of these groups demonstrated a mean BMI above 30 kg/m^2^, which is classified as obesity. The distribution of the Black women's BMI is displayed in Table 1, with the percentage of obese women in the DG being double that of the NDG. The percentage of overweight women in the NDG was treble that of the DG, and 24% were in the normal category in the NDG as compared with the DG (1%). The percentage of overweight and obese Black women attending the NCD clinic was 99% in the DG and 75% in the NDG. Only 11% of the DG women had a BMI of less than 30 kg/m^2^ compared with 56% in the NDG. One quarter of the NDG had a normal weight or were underweight, compared with only 1% in the DG, supporting the overall lower weight measurements amongst the NDG participants.

**TABLE 1:** Body Mass Index distribution of Black women with and without Type 2 Diabetes mellitus.

Weight categories	BMI kg/m^2^	DG		NDG	
			
		*n*	%	*n*	%
Underweight	< 18.5	0	0	1	1
Normal weight	18.5–24.9	3	1	22	24
Overweight	25–29.9	24	10	28	31
Obese	≥ 30	210	89	40	44

**Total**	-	**237**	**100**	**91**	**100**

*Source:* Data collected during study

NDG, non-diabetes group; DG, diabetic group; BMI, body mass index.

In the DG, underperceiving their correct body image was shown by all weight categories (*p* < 0.05), with those being overweight and obese having the widest variation with respect to their actual BMI. The NDG followed a similar trend, with the exception of the normal weight group (*p* = 0.4886), with 50% perceiving themselves to be overweight and only 17% obese, compared with the actual values of 31% – 44% in their respective BMI categories (*p* < 0.05). The pattern of incongruence in the PBI versus BMI observed in both groups was similar for at least the overweight and obese categories, as presented in Table 2.

**TABLE 2:** Perceived Body Image related to Body Mass Index of Black women with and without Type 2 Diabetes mellitus.

Body image/BMI categories	DG			NDG		
		
	PBI (%)	BMI (%)	*p*-value	PBI (%)	BMI (%)	*p*-value
Underweight	4	0	0.0446*	14	1	0.0005*
Normal weight	12	1	0.0020*	20	24	0.4886
Overweight	53	10	0.0000*	50	31	0.0046*
Obese	31	89	0.0000*	17	44	0.0000*

**Total**	**100**	**100**	**-**	**100**	**100**	**-**

*Source:* Data collected during study

* Implies statistically significant at the 95% alpha level.

NDG, non-diabetes group; DG, diabetic group; BMI, body mass index; PBI, perceived body image.

In the DG, 50% preferred an overweight body image, 45% associated a healthy body image with a normal weight, and 99% associated being underweight with a body image of an HIV patient. In the NDG, 59% preferred an overweight body image, 39% associated a healthy body image with a normal weight, and almost all (99%) related being thin to having HIV. Table 3 highlights the similarities and differences between the two groups.

**TABLE 3:** Preferred body image, healthy body image and body image representing people infected with HIV as demonstrated by Black women with and without Type 2 Diabetes mellitus.

Body image categories	Preferred body image (%)		Healthy body image (%)		Body image of HIV (%)	
			
	DG	NDG	DG	NDG	DG	NDG
Underweight	4	7	15	9	99	99
Normal weight	43	30	45	34	0.5	1
Overweight	50	59	36	50	0.5	0
Obese	3	4	4	7	0	0

**Totals**	**100**	**100**	**100**	**100**	**100**	**100**

*Source:* Data collected during study NDG, non-diabetes group; DG, diabetic group; BMI, body mass index.

## Trustworthiness

This study was conducted as per protocol and the results as outlined are a true reflection of the findings.

## Discussion

In the 1990s it was thought that Black women in SA had benign or healthy obesity with no adverse consequences on their health, but recent studies contradicting this opinion now dominate the literature.^11^ This indicates that the consequences of obesity in Black women are the same as in any other race, with the development of T2DM being a major cause for concern.^7^ The European Union has stressed the significance of considering the ethnosocio-cultural, governmental and structural–physical impacts for the successful anticipation and curtailing of the obesity pandemic and its NCD sequel.^17, 18^ The determinants of the SA obesity epidemic and the resultant development of T2DM are complex, with a Black women's self-perception attracting attention as a risk factor for obesity against a backdrop of high HIV burden, particularly in KZN.^13, 14^

This study aimed to compare the perception of body image of Black women with and without T2DM and to determine the degree of incongruence that exists in both these groups in relation to the measured BMI. The frequency of overweight and obese participants in the DG was 89% and for the NDG, 75%, which was higher than the national statistics reported in the SADHS^7^; however, as these women were recruited from an NCD clinic, this was to be expected. Both of these groups, however, face health challenges associated with excess weight, with the overburdened public health system also having to provide services to manage their T2DM and any secondary complications that may arise therefrom.^7^ The South African Nurse's Health Study showed that the risk of diabetes increased exponentially with a BMI above 22 kg/m^2^, indicating that the NDG participants who are overweight or obese are at risk of becoming new T2DM cases. In both groups, excess weight adds further strain to an already constrained health budget.^10, 11^

Bays et al.,^15^ on secondary examination of the ‘Study to Help Improve Early evaluation and management of risk factors Leading to Diabetes’ (SHIELD), concluded that a misperception of one's body image was common amongst women in the study and may have preceded the development of clinical diabetes.^15^ In the SA context, a Black women's perception of her body image may be considered as a possible barrier to weight loss which could help fuel the existing obesity epidemic.^6, 16^ This study showed that against a milieu of high HIV prevalence in KZN (16%), urban Black women with underlying NCDs misperceived their body image with a shift toward underestimating their body size across all weight categories. In the DG, only 31% correctly perceived themselves as being obese, although 89% had a BMI ≥ 30 kg/m^2^, with the NDG following a parallel pattern. These findings are similar to that reported by Puoane et al.^12^ amongst female community health care workers in Cape Town who, despite pre-existing knowledge of the consequences of excess weight, displayed incongruence between PBI and actual BMI, as well as resistance to weight loss for the fear of HIV stigmatisation, thereby compromising their position as educators.^16, 17^

Of particular worry is the fact that the majority of Black women in both groups in this study preferred an overweight body image. In contrast, Matoti-Mvalo and Puoane^14^ reported that in Khayelitsha, Cape Town, 50% of the urban Black women favoured a normal weight over the overweight and obese body images. This difference could possibly be attributed to the lower HIV prevalence in this district of Cape Town (6%) as compared with KZN (16%) where the HIV sensitisation is greater.^14, 17^ As a further complication, the findings in the ‘Big is Beautiful’ study in Khayelitsha, Cape Town, which concluded that being rounded in body shape had many positive connotations (amongst others to increase their desirability for men), further add to the complexity in addressing the obesity epidemic in the SA context where strategies to engage such women to lose weight may be futile, thereby perpetuating the vicious cycle of weight gain and its sequel.^16, 23, 24^

Almost half the Black women with pre-existing DM (45%) in this study correctly associated a healthy body image with normal weight. However, 50% of the NDG linked the overweight body image to health and freedom from disease, compared with 34% of Black urban women in Khayelitsha, Cape Town, as reported by Matoti-Mvalo and Puoane^14^, who attached the healthy body image to the normal weight category.^14^ It could be speculated that the Black women in the NDG may be more predisposed to clinical diabetes owing to a difference in perception, as they may not find it necessary to engage in a weight loss management programme, thereby fuelling the overweight and obesity statistics reported by the SADHS.^7^ Similarly, the DG faces additional health challenges since although most women in the DG appropriately linked a healthy body image and weight, more than a third (36%) also linked an overweight body image to being healthy and free of disease, whilst most women in this group (50%) personally preferred a larger body size.

The high level of incongruence in PBI and BMI as demonstrated in this study in both the DG and NDG might be linked to the rising HIV burden in the KZN province (16%), seeing as almost all of the Black women in both the groups strongly linked being thin to having HIV. Matoti-Mvalo and Puoane showed similar, but somewhat lower, figures amongst Black women from Khayelitsha, Cape Town, where 70% of the participants believed that being thin is an indication of HIV infection.^14^ The lower percentage of women who linked being underweight to having HIV is possibly due to the lower prevalence of HIV disease (6%) in this area.^17^

## Study limitations and recommendations

A limitation in this study was that the HIV status of the individuals was not requested; however, the findings do imply that there is a high level of sensitisation regarding HIV infection in the area. Furthermore, the findings cannot be extrapolated to other race groups for it has been shown that women of different racial backgrounds differ in their perception of their body image, which opens another avenue for further research. The study participants were urban Black women recruited from an NCD clinic and they may thus differ from the general population; future studies should therefore encompass a greater population radius in order to be more representative.

## Conclusion

This study demonstrated a high occurrence of BMIs above 25kg/m^2^ amongst Black urban women with NCD, putting those with pre-existing T2DM at a higher risk of developing secondary diabetes-related complications associated with excess weight, and putting those without T2DM at risk of developing clinical T2DM. The patterns of incongruence in PBI and BMI were similar in both groups and, against a backdrop of high HIV prevalence, both groups showed an almost unanimous perception that being thin is associated with HIV.

The risk factors for T2DM which need to be addressed in the South African context should be broadened to include incongruence in PBI and BMI as being a surrogate risk marker. The results indicate that weight loss strategies for those women therefore need to be ethnosocio-culturally specific in order to be both acceptable and successful. Furthermore, clinicians need to be aware of the fact that HIV stigmatisation may be altering perceptions and therefore behaviour, in order to better phrase their recommendation for the need for weight reduction.
